# Efficacy and safety of a bridging strategy that uses intravenous platelet glycoprotein receptor inhibitors for patients undergoing surgery after coronary stent implantation: a meta-analysis

**DOI:** 10.1186/s12872-022-02563-3

**Published:** 2022-03-24

**Authors:** Fan Wu, Kanghua Ma, Rui Xiang, Baoru Han, Jing Chang, Zhong Zuo, Yue Luo, Min Mao

**Affiliations:** 1 Department of Cardiology, The First Affiliated Hospital of Chongqing Medical University, No. 1, Youyi Road, Yuanjiagang, Yuzhong District, Chongqing, 400016 China; 2 College of Medical Informatics, Chongqing Medical University, Chongqing, 401135 China

**Keywords:** Antiplatelet therapy, Bridging therapy, Tirofiban, Eptifibatide, Surgery

## Abstract

**Background:**

Current guidelines indicate we can consider a bridging strategy that uses intravenous, reversible glycoprotein inhibitors for patients that required surgery following recent stent implantation. However, no strong clinical evidence exists that demonstrates the efficacy and safety of this treatment. Therefore, in this study, the efficacy and safety of a bridging strategy that uses intravenous platelet glycoprotein receptor inhibitors will be evaluated.

**Methods:**

A meta-analysis was performed on preoperative bridging studies in patients undergoing coronary stent surgery. The primary outcome was the success rate of no major adverse cardiovascular events (MACE). The secondary outcomes were the success rate of no reoperations to stop bleeding.

**Results:**

A total of 10 studies that included 382 patients were used in this meta-analysis. For the primary endpoint, the success rate was 97.7% (95% CI 94.4–98.0%) for glycoprotein IIb/IIIa inhibitors, 98.8% (95% CI 96.0–100%) for tirofiban (6 studies) and 95.8% (95% CI 90.4–99.4%) for eptifibatide (4 studies). For secondary endpoints, the success rate was 98.0% (95% CI 94.8–99.9%) for glycoprotein IIb/IIIa inhibitors, 99.7% (95% CI 97.1–100%) for tirofiban (5 studies), and 95.3% (95% CI 88.5–99.4%) for eptifibatide (4 studies).

**Conclusion:**

The results of this study showed that the use of intravenous platelet glycoprotein IIb/IIIa inhibitors as a bridging strategy might be safe and effective for patients undergoing coronary stent implantation that require surgery soon after.

## Background

Current guidelines recommend that patients diagnosed with stable coronary artery disease with stent implants should receive dual antiplatelet therapy (DAPT) that uses a P2Y12 inhibitor and aspirin for 6 months and patients with acute coronary syndrome for 12 months unless they show contraindications such as bleeding [1–6]. However, approximately 5% of patients undergo surgery in the first year following their percutaneous coronary intervention (PCI) [7–9]. Dual antiplatelet therapy increases the intra and perioperative bleeding risk, and surgery is associated with pro-inflammatory and pro-thrombotic effects; therefore, increasing the risk of coronary thrombosis at the level of the stented vascular segment and throughout the coronary vasculature [10].

Recent guidelines indicate that intravenous antiplatelet drugs may be considered for perioperative bridging treatment. However, to the best of the authors’ knowledge, there is no strong clinical evidence that demonstrates the efficacy of bridging with either parenteral antiplatelet therapies [1, 3]. Therefore, in this meta-analysis, the efficacy and safety of a bridging strategy that uses intravenous platelet glycoprotein receptor inhibitors will be evaluated.

## Methods

A systematic search was conducted to identify relevant studies within databases, such as PubMed (January 1, 1946, to November 15, 2020), EMBASE (January 1, 1974, to November 15, 2020), and the Cochrane Library (inception to November 15, 2020). As a result, the following keywords were used: antiplatelet therapy, aspirin, clopidogrel, ticagrelor, prasugrel, GP IIb/IIIa inhibitor abciximab, tirofiban, eptifibatide, and surgery.

Any experimental and observational studies were included except for case reports without any limits and language restrictions. These studies described the use of an intravenous antiplatelet bridging treatment strategy for patients with coronary heart disease that had a stent implanted within 6 months of when surgery was planned.

Two reviewers independently extracted the data and contacted the relevant authors to obtain detailed data when the information was not comprehensive. Disagreement was resolved through negotiation. In addition, when there was no consensus, a recommendation from a third reviewer was involved.

Finally, 18 evaluation checklists were formed based on improvements to the Delphi technology. They were used to evaluate the quality of the case series study methodology. Each study counted the total number of positive items based on the consensus of the reviewers. This methodological quality assessment checklist did not recommend using scoring methods but gave corresponding options for each item. If the study met 14 (70%) or more checklist parameters, it was considered acceptable.

The primary outcome of this meta-analysis was the success rate of no major adverse cardiac events defined by each study, such as myocardial infarction, stent thrombosis, cardiogenic shock, sudden cardiac death, and death. The secondary outcome was the success rate of no reoperation to stop bleeding as defined by each study.

Statistical analysis was performed using Stata (Stata15, USA) software. To calculate the success rate of the bridging treatment, calculations were based on two aspects, avoiding major adverse cardiovascular events (primary endpoint) and avoiding reoperation due to bleeding (secondary endpoint). Cochrane Q statistic (*p*-values ≤ 0.1 were considered significant) and I^2^ statistic (25%, 50%, and 75% were associated with low, medium, and high heterogeneity, respectively) were used to evaluate the heterogeneity between various studies. Publication bias was assessed using Egger's test (*p* < 0.1). In addition, the sensitivity was evaluated to ensure the robustness of the results, and subgroup analysis was conducted after the bridge therapy drugs were separated (tirofiban and eptifibatide).

## Results

**Fig. 1:**
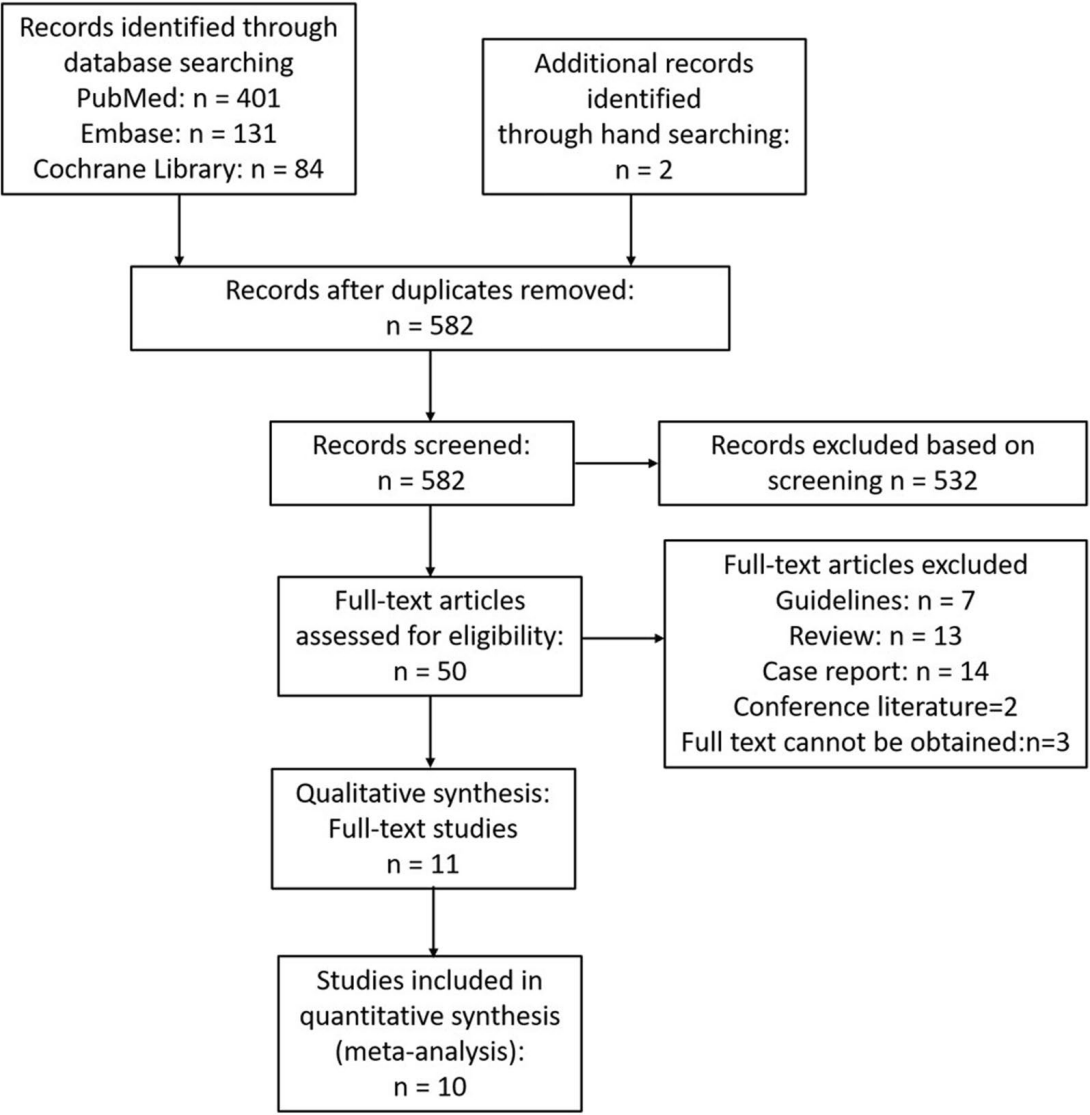
Transparent reporting of meta-analyses flow diagram outlining the search strategy results from initial search to included studies

The initial search yielded 582 unique studies for review (Fig. 1). Following the screening, a full-text review was conducted on 50 special reports. Finally, 11 studies were included in the qualitative synthesis. Out of 11 studies, 10 provided sufficient details for meta-analysis. The key characteristics and findings of the included studies, which included two prospective and eight observational studies, are given in Table 1. Other important details of each article are shown in Table 2.

**Table 1:** Key characteristics and findings of the included studies

References	Country	No. of patients	Age	Type	Non-cardiac surgery(n)	Type of stents	Time from PCI to surgery (months)	Bridging protocol	Non-MACE		Non-reoperation	
									Number	Success rate(95% CI)	Number	Success rate(95% CI)
Xia (2013)	China	21	63	Obs,Prosp	21(100%)	DES	6	Initiated tirofiban 4.8 days prior to surgery; stopped tirofiban 4 h prior to surgery	21	100% (83.9–100%)	21	100% (83.9–100%)
Walker (2017)	USA	20	72	Obs,Restrosp	11(55%)	DES	1.1	Initiated tirofiban 3.5 days prior to surgery; stopped tirofiban 5.4 h prior to surgery	18	90.0% (68.3–98.8%)	20	100% (83.2–100%)
Savonitto (2010)	USA	30	65	Obs,Prosp	21(70%)	DES	4	Initiated tirofiban 4 days prior to surgery; stopped tirofiban 5 h prior to surgery	30	100% (88.4–100%)	30	100% (88.4–100%)
Polito (2018)	Italy	21	62	Obs,Restrosp	0(0%)	DES	0,23	Initiated tirofiban 3 days prior to surgery; stopped tirofiban 4 h prior to surgery	20	95.2% (76.2–99.9%)	21	100% (83.9–100%)
Marcos (2011)	Netherlands	36	66	Obs,Restrosp	21(58%)	Mixed(58%DES)	2.6	Initiated tirofiban 4 days prior to surgery; stopped tirofiban 4 h prior to surgery	36	100% (90.3–100%)	35	97.2% (85.5–99.9%)
Servi (2016)	Italy	87	67.4	Obs,Restrosp	59(68%)	91%	3.4	Initiated tirofiban 3 days prior to surgery; stopped tirofiban 4 h prior to surgery	85	97.7% (91.9–99.7%)	87	100% (95.8–100%)
Waldron (2017)	USA	30	65	Obs,Restrosp	30(100%)	DES	Not mention	Used eptifibatide, not mention bridging details	27	90.0% (73.5–97.9%)	30	100% (88.4–100%)
Rassi (2012)	USA	100	63.2	Obs,Restrosp	29(29%)	Mixed(89%DES)	5.8	Initiated eptifibatide 5.3 days prior to surgery; stopped eptifibatide 7 h prior to surgery	93	93.0% (86.1–97.1%)	90	90.0% (82.4–95.1%)
Morrison (2012)	USA	19	65	Obs,Restrosp	6(32%)	DES	3.5	Initiated eptifibatide 2.6 days prior to surgery; stopped eptifibatide 10 h prior to surgery	19	100% (82.4–100%)	18	94.7% (74.0–99.9%)
Barra (2016)	USA	18	61.9	Obs,Restrosp	13(72%)	Mixed(89%DES)	3.5	Initiated eptifibatide 2.7 days prior to surgery; stopped eptifibatide 6 h prior to surgery	18	100% (81.5–100%)	17	94.4% (72.7–99.9%)

**Table 2:** Key information of the included studies

Reference	No. of patients	MACE definition	The follow-up time	DAPT (No. of patients)	Dose for bridging therapy
Xia (2013)	21	Cardiovascular death, MI, target lesion revascularization	3 months	Aspirin(21) Clopidogrel(21)	0.1 µg/kg/min 0.05 µg/kg/min(if CrCl < 30 mL/min)
Walker (2017)	20	Death,repeat myocardial infarction, stent thrombosis,or target lesion revascularization	During hospitalization	Aspirin(20) Clopidogrel(16) Ticagrelor(4)	0.1 µg/kg/min 0.05 µg/kg/min(if CrCl < 30 mL/min)
Savonitto (2010)	30	Cardiovascular death, MI, an acute occlusion of the target lesion	During hospitalization	Aspirin(30) Clopidogrel(30)	0.1 µg/kg/min 0.05 µg/kg/min(if CrCl < 30 mL/min)
Polito (2018)	21	Death, reinfarction, cardiovascular events	21.6 months	Aspirin(21) Clopidogrel(1) Ticagrelor(13) Prasugrel(7)	0.1 µg/kg/min 0.05 µg/kg/min(if CrCl < 30 mL/min)
Marcos (2011)	36	Death, repeat myocardial infarction, target vessel revascularisation, target lesion revascularization, stent thrombosis	30 days	Aspirin(36) Clopidogrel(36)	Not mentioned
Servi (2016)	87	All-cause death; myocardial infarction; definite stent thrombosis	30 days	Aspirin(87) Clopidogrel(84) Ticagrelor(1) ticlopidine (2)	0.1 µg/kg/min 0.05 µg/kg/min(if CrCl < 30 mL/min)
Waldron (2017)	30	Myocardial infarction or death	30 days	Aspirin(30) Clopidogrel(30)	2 µg/kg/min 1 µg/kg/min(if CrCl < 30 mL/min)
Rassi (2012)	100	Death, myocardial infarction, urgent revascularization, and ischemic stroke	During hospitalization	Aspirin Clopidogrel prasugrel (No specific number)	Not mentioned
Morrison (2012)	19	Stent thrombosis acute coronary syndrome, and death	30 days	Aspirin(19) Clopidogrel(19)	2 µg/kg/min 1 µg/kg/min(if CrCl < 30 mL/min)
Barra (2016)	18	Stent thrombosisand death	90 days	Aspirin(18) Clopidogrel(15) Prasugrel(3)	2 µg/kg/min 1 µg/kg/min(if CrCl < 30 mL/min)

This study included ten studies [11–20] with 382 patients. Among them, 6 studies [11, 12, 14, 16–18] included 215 patients that used tirofiban for bridging therapy. Four studies [13, 15, 19, 20] had 167 patients that used eptifibatide for bridging therapy.

MACE was reported in all studies included, while reoperation due to bleeding was reported in seven studies. Walker 2017 mentioned that four bleeding events occurred in all patients, three minimal TIMI bleeding, one minor TIMI bleeding, and only one required blood transfusion and drug discontinuation. The results of Polito 2018 suggested three cases of uncomplicated anemia in bridging patients after surgery. So we believed that there were no cases of reoperation due to bleeding in these studies and included them in the calculation. Reoperation due to bleeding might be considered the most objective safety endpoint because it does not depend on the different criteria adopted for bleeding transfusion. All studies included were supposed to be of acceptable quality according to the modified Delphi technique (Table 3) [21].

**Table 3:** Quality assessment checklist

18-Criteria checklist	Number of studies saying yes	Number of studies saying no
1. Is the hypothesis/aim/objective of the study stated clearly in the abstract, introduction, or methods section?	10	0
2. Are the characteristics of the participants included in the study described?	10	0
3. Were the cases collected in more than one center?	2	8
4. Are the eligibility criteria (inclusion and exclusion criteria) for entry into the study explicit and appropriate?	10	0
5. Were participants recruited consecutively?	7	3
6. Did participants enter the study at a similar point in the disease?	8	2
7. Was the intervention clearly described in the study?	10	0
8. Were additional interventions (co-interventions) clearly reported in the study?	10	0
9. Are the outcome measures clearly defined in the introduction or methods section?	10	0
10. Were relevant outcomes appropriately measured with objective and/or subjective methods?	9	1
11. Were outcomes measured before and after intervention?	8	2
12. Were the statistical tests used to assess the relevant outcomes appropriate?	7	3
13. Was the length of follow-up reported?	5	5
14. Was the loss to follow-up reported?	10	0
15. Does the study provide estimates of the random variability in the data analysis of relevant outcomes?	8	2
16. Are adverse events reported?	10	0
17. Are the conclusions of the study supported by results?	10	0
18. Are both competing interests and sources of support for the study reported?	10	0

**Fig. 2:**
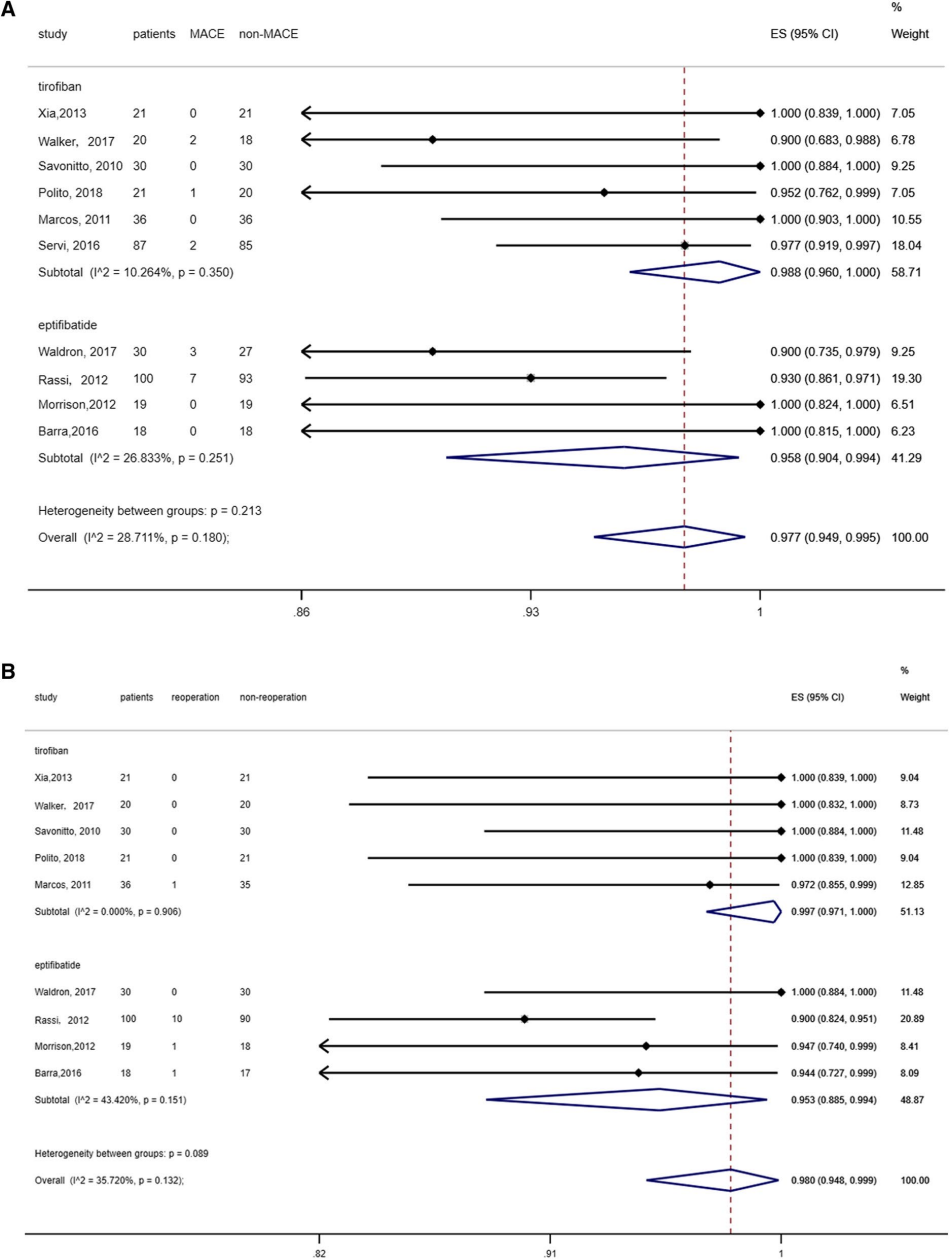
**a** The forest map for non-MACE, **b** the forest map for non-reoperation

**Fig. 3:**
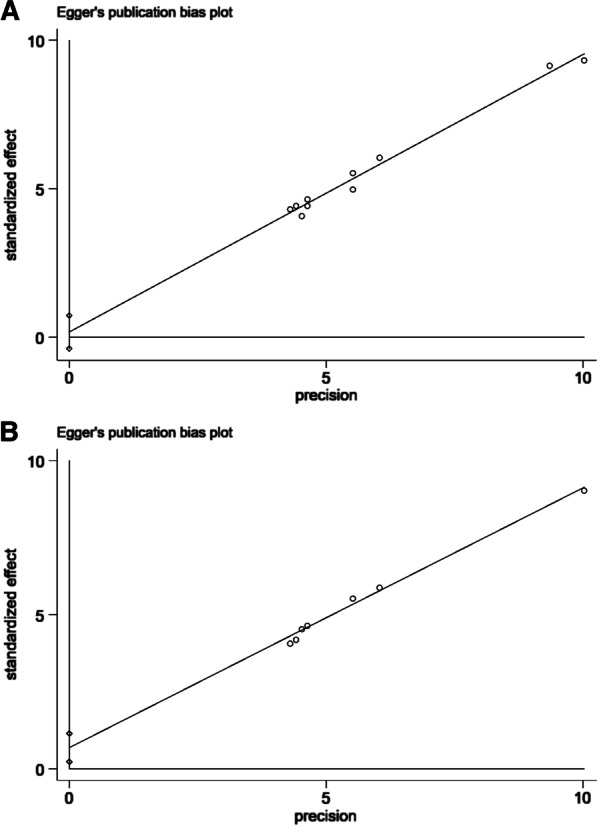
**a** The funnel plot for non-MACE, **b** the funnel plot for non-reoperation

**Fig. 4:**
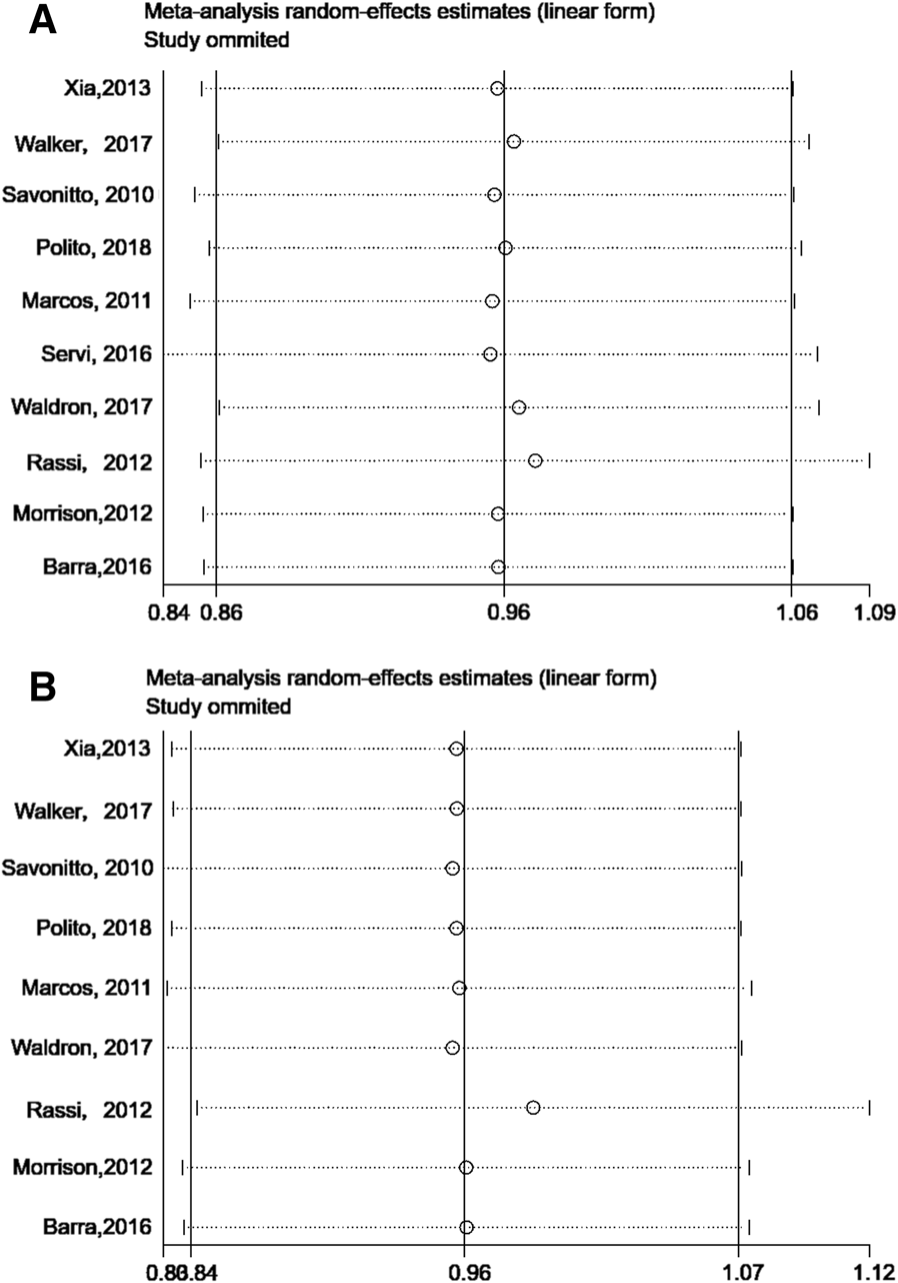
**a** The sensitivity analysis for non-MACE, **b** the sensitivity analysis for non-reoperation

Among the 382 patients, 367 did not show any major adverse cardiac events, and the success rate was 97.7% (95% CI 94.4–98.0%). According to the results of six studies, the success rate of tirofiban was 98.8% (95% CI 96.0–100%). In addition, the success rate of eptifibatide was 95.8% (95% CI 90.4–99.4%) based on the results of four studies (Fig. 2a). The risk of publication bias appeared to be low (*p* = 0.508, 95% CI − 0.391 to 0.727) (Fig. 3a). The findings were robust to sensitivity analyses performed for bias, study design, type of operation, and the variations in estimate modeling mentioned previously (Fig. 4a).

Because each study used a different definition of bleeding, and each patient underwent another kind of surgery, the risk of bleeding differed. Therefore, reoperation due to bleeding was considered the secondary endpoint of the study. Among the 382 patients, 369 did not record reoperation due to bleeding, and the success rate was 98.0% (95% CI 94.8–99.9%). According to the results from six studies, the success rate of tirofiban was 99.7% (95% CI 97.1–100%). The success rate of eptifibatide was 95.3% (95% CI 88.5–99.4%) based on the results of four studies (Fig. 2b). The risk of publication bias appeared to be low (*p* = 0.11, 95% CI − 0.227 to 1.145) (Fig. 3b), and sensitivity analysis was robust (Fig. 4b).

## Discussion

This study showed that the use of glycoprotein IIb/IIIa inhibitors for bridging antiplatelet therapy might be safe and effective for patients undergoing coronary stent implantation that require surgery within 6 months and whose bleeding was classified as high risk. However, the intensive care unit must perform the bridging treatment with sufficient monitoring and testing conditions.

Glycoprotein IIb/IIIa are receptors on the surface of platelets that mediate the binding of fibrinogen, von Willebrand factor, and vitronectin to platelets, which cause platelets to cross-link and aggregate. Abciximab, tirofiban, and eptifibatide block this process in a targeted manner. Tirofiban and eptifibatide have a shorter action time, and their platelet inhibitory effect can last for 2–4 h following administration [22].

The previous studies [23] found that in patients with coronary heart disease that underwent non-cardiac surgery within 1 month of coronary stent implantation, the incidence of perioperative death, acute myocardial infarction, stent thrombosis, and other cardiac adverse events was as high as 30%; in 2 out of 6 of cases, surgery was performed at the end of the month, and the incidence of the previously mentioned adverse events decreased to 10–15%; after 6 months, the incidence of surgery decreased to < 10%. Therefore, for patients with coronary stent implantation, if surgery with a high risk of bleeding is planned, it is necessary to carefully assess the advantages and disadvantages (e.g., the risk of cardiovascular complications that are caused by discontinuing antiplatelet drugs and the risk of bleeding caused by continuing drugs).

According to a multidisciplinary management opinion [24], in patients > 12 months after PCI, the risk of perioperative thromboembolism was low, and therefore, elective surgery could be performed. In patients < 12 months after PCI, the time for elective operation should be determined based on several factors. In summary, elective surgery should be performed ≥ 2 weeks after coronary artery balloon dilation, ≥ 1 month after implantation of a BMS, and ≥ 3 months after the implantation or elective surgery is performed again. For the new generation of DES, the time could be appropriately shortened based on the situation, and elective surgery should be performed ≥ 12 months after implantation of a bioabsorbable stent (BVS).

The risk of bleeding during the perioperative period is mainly affected by the type of surgery or the invasive nature of the procedure. In general, any long-duration (> 45 min) surgery and surgery on vital organs (e.g., central nervous system and heart), blood-rich organs (e.g., liver and spleen), large blood vessels, active fibrinolytic sites (i.e., the urinary system) and invasive procedures should be considered to have a high risk of bleeding [25, 26]. In patients treated with antiplatelet drugs before surgery, the PRECISE‑DAPT score could be used to evaluate the patient’s risk of bleeding [27].

This study has two main limitations. First, the included studies were observational and were not randomized controlled experiments. Second, the included studies used a different definition of bleeding; therefore, only the success rate of freedom from reoperation without bleeding was included.

## Conclusions

In patients that require surgery after recent stent implantation, a bridging strategy that uses intravenous platelet glycoprotein receptor inhibitors might allow the temporary discontinuation of dual antiplatelet drugs without increasing the risk of bleeding. The decision to perform bridging treatment and careful risk stratification of ischemic events and bleeding requires strict cooperation between surgeons, cardiologists, and anesthesiologists. Large-scale randomized clinical trials are needed to confirm this result further.

## Data Availability

The datasets used and/or analyzed during the current study are available from the corresponding author on reasonable request.

## Abbreviations

MACE: Major adverse cardiovascular events
BMS: Bare-metal stent
DES: Drug-eluting stent
DAPT: Dual antiplatelet therapy
PCI: Percutaneous coronary intervention
BVS: Bioabsorbable stent
